# Short stem hip arthroplasty with the optimys prosthesis is a safe and effective option for obese patients: a mid-term follow-up multicenter study

**DOI:** 10.1007/s00402-023-05105-4

**Published:** 2023-11-04

**Authors:** Nico Hinz, Giulia Marsoni, Hagen Mittelstädt, Falk Sonnabend, Karsten Wallroth, Carsten Johl, Ulrich Weigert, Conrad Anderl, Reinhold Ortmaier, Natividad Zeleny, Arndt-Peter Schulz

**Affiliations:** 1 BG Hospital Hamburg, Trauma Surgery, Orthopedics and Sports Traumatology, Bergedorfer Strasse 10, 21033 Hamburg, Germany; 2 Erzgebirgsklinikum, Orthopedics and Trauma Surgery, Jahnsdorfer Strasse 7, 09366 Stollberg, Germany; 3 University Medical Center Schleswig-Holstein, Campus Lübeck, Orthopaedic and Trauma Surgery, Ratzeburger Allee 160, 23538 Lübeck, Germany; 4 Helios Klinik Köthen, Orthopedics, Hallesche Strasse 29, 06366 Köthen, Germany; 5 Klinikum Dahme-Spreewald, Orthopedics and Trauma Surgery, Schillerstrasse 29, 15907 Lübben, Germany; 6 Practice for Orthopedics and Trauma Surgery, Friedrichstrasse 1-3, 15537 Erkner, Germany; 7 Ordensklinikum Linz Barmherzige Schwestern, Orthopedics, Seilerstätte 4, 4010 Linz, Austria; 8 Mathys Orthopedics GmbH, Seilfahrt 99, 44809 Bochum, Germany; 9 Medical Faculty, Universität zu Lübeck, Ratzeburger Allee 160, 23562 Lübeck, Germany

**Keywords:** Short stem THA, Optimys prosthesis, Obesity, Harris Hip Score

## Abstract

**Introduction:**

Short stems are a valuable option in young patients undergoing total hip arthroplasty (THA) because of their bone stock preserving properties facilitating revision hip arthroplasty. Although the effect of obesity on conventional THA is well studied, data about short stem THA in obese patients are lacking. Therefore, this study aimed to investigate the influence of obesity on complications, revisions, and outcome after short stem THA.

**Materials and methods:**

This multicenter, observational cohort study included patients undergoing short stem THA with the optimys prosthesis. Follow-up examinations were performed at specific intervals up to 7 years postoperatively. Operation characteristics, general and specific complications, revisions, VAS rest pain, VAS load pain, VAS patient satisfaction, and Harris Hip Score (HHS) were recorded and statistically compared between obese (BMI ≥ 30 kg/m^2^) and non-obese (BMI < 30 kg/m^2^) patients.

**Results:**

Of the 224 patients included with a mean follow-up of 87.2 months (range 81.9–104.0), 69 were assigned to the OB group and 155 to the non-OB group. A minimally invasive approach was significantly less often selected in obese patients (*p* = 0.049), whereas operating time and length of hospital stay were not significantly different. The rate of general and specific complications did not significantly differ between both groups. Survival of the optimys prosthesis was 99.1% at 7-year follow-up and one patient per group had to undergo revision surgery. VAS rest pain, load pain, and satisfaction improved from preoperatively to postoperatively in both groups without a significant difference between both groups. While the HHS was improved from preoperatively to postoperatively, obese patients showed a significantly lower HHS at the 7-year follow-up (*p* = 0.01) but still exhibited an excellent scoring above the PASS threshold.

**Conclusion:**

Short stem THA with the optimys prosthesis is a safe and effective option also in obese patients with an excellent clinical outcome and a low complication rate.

## Introduction

Due to an aging society, the number of patients, who need a total hip arthroplasty (THA), increased considerably over the past years and is estimated to increase further in the future [1–3]. Furthermore, the proportion of patients, who require THA at younger age and at a more physically active stage of live, is growing [4–7]. Because of an average survival rate of a primary THA of about 58% after 25 years, these younger patients are statistically at a high risk for at least one revision hip arthroplasty in their lifetime [6, 8–10]. Bone stock preserving implants for primary THA are, therefore, required to maintain a sufficient bone stock for revision hip arthroplasty.

In recent years, short stem THA became a popular option in addition to THA with conventional prosthesis stems, especially in young and active patients, with excellent clinical results [11, 12]. The major advantage of short stem prostheses and the cause of their frequent use in young patients is to provide a favorable condition for revision hip arthroplasty when explanted, because of their bone- and soft-tissue-sparing implantation as well as prevention of stress shielding and periprosthetic bone loss due to a more physiological load transfer [11–14]. The short stem prosthesis optimys from Mathys is one of the newest generation among the large variety of short stem protheses and is particularly bone- and soft-tissue sparing as well as provides an enhanced primary stability and osseointegration [11, 15–18].

Obesity, defined as a BMI ≥ 30 kg/m^2^, is a rising health problem worldwide and the prevalence increased from 3.2 to 10.8% in men and from 6.4 to 14.9% in women between 1975 and 2014 [19]. Obesity is a well-identified risk factor for the development of hip osteoarthritis and thus, the proportion of patients with obesity, who receive a THA, is increasing [20–23]. Obesity was shown to be a risk factor for complications, especially infections and dislocations, after THA as well as for revision surgeries [24–29]. Data about the functional outcome after THA in obese patients are inconclusive but tend not to show a significant difference compared to non-obese patients, except of the Harris Hip Score showing lower scores for obese patients in some studies [25, 26, 28–30]. Moreover, obese patients undergo THA significantly earlier than non-obese patients, and therefore belong to the above-described group of patients, where bone-sparing implants for primary THA are favorable because of statistically at least one revision hip arthroplasty in their lifetime [25, 31, 32].

However, there are only limited and partly inconclusive data about the effect of obesity in short stem THA, especially on mid-term and long-term outcomes [33–36]. This is why, the purpose of this study was to a investigate the effect of obesity in short stem THA with the optimys implant on operation characteristics, complications, revision surgeries, and clinical outcome, such as postoperative pain, patient satisfaction, and Harris Hip Score.

## Materials and methods

### Study design and study cohort

This study is part of the multicenter optimys study, which is an ongoing, post-market clinical follow-up, prospective, observational cohort study of patients receiving an optimys short stem. This report follows the STROBE guidelines [37]. The patient recruitment period began on February 15, 2012 and ended on September 24, 2013. In this period, 224 patients were included in 6 study centers according to the following inclusion criteria:

1. Patients suffering from primary or secondary hip osteoarthritis due to the following pathologies: coxarthrosis, dysplastic coxarthrosis, rheumatoid arthritis, femoral head necrosis or posttraumatic coxarthrosis.
2. Patients being a candidate for primary THA
3. Patients receiving a short stem THA with the optimys prosthesis
4. Patients between the age of 18 and 85 years at the time of inclusion
5. Patients being expected to recover completely
6. Patients willing to participate in this study and the follow-up examinations, being able to understand the character of this study and providing written informed consent

The following exclusion criteria were followed:

1. Patients undergoing revision hip arthroplasty
2. Patients suffering from sepsis or malignant tumors
3. Patients having an ASA classification > 3
4. Patients being not able to participate in the regular follow-up examinations
5. Patients simultaneously participating in another clinical study or documentation with other orthopedic implants of other manufacturers

The indication to perform a short stem THA using the optimys prosthesis was made by the patient’s treating physicians independently of this study.

Each patient was screened directly preoperatively and after informed consent was obtained, preoperative data acquisition including demographic data, preoperative patient’s characteristics, VAS pain, VAS satisfaction, Harris Hip Score, and preoperative planning X-rays was performed. During the index hospitalization, data about the surgery and short-term complications until discharge were recorded. Subsequently, follow-up visits were performed after 6 weeks–3 months, 1 year, 2 years, 5 years, and 7 years postoperatively. Each follow-up visit included recording of complications occurred, VAS pain, VAS satisfaction, Harris Hip Score, and X-ray.

Patients were excluded from further follow-up, if one of the following dropout criteria was met:

1. Death of the patient
2. Explantation of the optimys short stem
3. Loss to follow-up (withdrawal to participate in the study or loss for other reasons)

For the purpose of this study, the included patients were retrospectively assigned to the following two groups based on the BMI at the time of surgery: obese patients with a BMI ≥ 30 kg/m^2^ (OB group) and non-obese patients with a BMI < 30 kg/m^2^ (Non-OB group).

The study was conducted according to the ethical standards of the 1964 Declaration of Helsinki, the GCP standards and was approved by the local ethics committee (reference number: 12-112).

### Surgical technique and perioperative management

Each patient underwent THA with the uncemented short stem optimys because of a primary or secondary hip osteoarthritis. A modular acetabular cup or a monoblock acetabular cup with or without cement was implanted according to the surgeon’s choice. Prosthesis heads either made of aluminum oxide ceramic or made of dispersion ceramic were used. The surgical approach (direct anterior, anterolateral, lateral) was chosen by the surgeon and the assessment of whether a minimally invasive or a non-minimally invasive approach was used, was reported by the surgeon after implantation.

Intraoperatively, patients received a single shot antibiosis. Postoperatively, a thrombosis prophylaxis for at least 28–35 days and analgesia as needed were applied. Patients received physiotherapy and were mobilized with complaint-adapted full weight-bearing starting from the day after surgery.

### Clinical data and scores

During preoperative data acquisition, the following demographic data and patient’s characteristics were recorded: height, weight, BMI, age, sex, indication for THA, Charnley classification [38, 39] and previous surgeries at the affected hip. During the surgical implantation, the following data were collected: side of implantation, operating time, surgical approach used. Furthermore, the surgeons had to determine after the surgery whether they had used a minimally invasive or a non-minimally invasive approach. Postoperatively, the length of hospital stay, occurrence, and type of general and specific complications and performing of revision surgeries were recorded in each follow-up examination. Preoperatively and at each follow-up examination, the patients had to indicate the intensity of rest pain and load pain as well as their overall satisfaction with the VAS scale (0 = no pain or completely unsatisfied; 10 = strongest pain imaginable or completely satisfied). The Harris Hip Score (HHS) was surveyed preoperatively and at each follow-up examination to evaluate the clinical outcome [40]. The following grading of the HHS was applied in this study: < 70 = poor, 70–79 = fair, 80–89 = good, 90–100 = excellent [41]. In addition, the HHS was compared to the recently validated threshold for a patient acceptable symptom state (PASS) for each follow-up visit [42]. Axial migration of the short stem prosthesis was assessed with the validated femoral component analysis using Einzel-Bild-Röntgen-Analyse (EBRA-FCA) using pelvic X-rays from postoperative visits [43].

### Statistical analysis

Sample size calculation with G*Power 3.1 software revealed a sample size of at least 64 patients per group (calculation for HHS and VAS pain considering an independent two-tailed *t* test with effect size *d* = 0.5; *α* = 0.05; power 0.8; allocation rate N1/N2 = 1).

The survival rate of the optimys short stem was calculated using the following equation:

$${\text{Survival}} = \frac{{\left( {{\text{number}}\;{\text{of}}\;{\text{patients}} - {\text{number}}\;{\text{of}}\;{\text{revisions}}} \right)}}{{{\text{number}}\;{\text{of}}\;{\text{patients}}}} \times 100.$$

The following equation was used to calculate the standardized revision rate as specified by van Oladenrjik [44]:

$${\text{Revision }}\;{\text{rate}} = \frac{{{\text{number}}\;{\text{of}}\;{\text{revisions}}}}{{{\text{number}}\;{\text{of}}\;{\text{patients}}\; \times \;{\text{years}}\;{\text{of}}\;{\text{follow up}}}} \times 100.$$

A benchmark for the standardized revision rate per 100 component years of less than 1 was used in this study to consider the optimys short stem as safe and effective [15].

Missing values in the items of the HHS were handled as described in the scoring manual. Other missing data were handled by listwise deletion. Patients, who presented with a dropout criteria during one of the follow-up visits, were not further considered in the subsequent follow-up visits.

Data analysis and visualization were performed using GraphPad Prism 9 (GraphPad Software, La Jolla, CA, USA). Normal distribution of data was tested using the Shapiro–Wilk test. Statistical comparison of mean values between the two groups was performed using Student’s *t* test or Mann–Whitney-*U*-test for parametric or non-parametric data, respectively. If categorical data or frequencies were compared, Chi-squared test was used. Data are presented as mean ± standard deviation (SD) unless otherwise stated. *p* values of 0.05 or less were considered statistically significant and exact *p* values are reported unless *p* < 0.001. Analysis of the axial migration using EBRA-FCA was only done descriptively according to the threshold of 1.5 mm indicating an increased risk for THA failure described by Krismer et al. [45].

## Results

### Patient characteristics

**Fig. 1:**
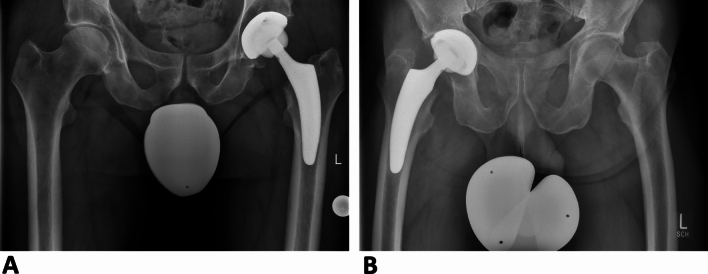
Representative pelvic X-rays of the 7-year follow-up exemplary for the OB group (**A**) and the Non-OB group (**B**)

A total of 224 patients, who received an optimys short stem were included in this study. Of these, 69 patients had a BMI of ≥ 30 kg/m^2^, and thus were assigned to the obese patient group (OB). On the other side, 155 patients with a BMI of < 30 kg/m^2^ were assigned to the non-obese control group (Non-OB). Figure 1 shows representative pelvic X-rays of the implanted short stem prosthesis in both groups.

Table 1 shows the demographics and clinical patient’s characteristics. The OB group had a significantly higher weight (98.4 ± 15.3 kg vs. 75.9 ± 12.3 kg; *p* < 0.001) and BMI (33.5 ± 2.8 kg/m^2^ vs. 25.9 ± 2.6 kg/m^2^; *p* < 0.001) compared to the Non-OB group. Apart from that, both groups did not significantly differ in terms of sex, age, height, and side of THA. In 63.8% of the OB group and in 68.4% of the Non-OB group, short stem THA was performed due to a primary hip osteoarthritis, whereas secondary hip osteoarthritis was the indication for 15.9% in the OB group and for 18.7% in the Non-OB group. 20.2% of the obese patients and 12.9% of the non-obese patients received a short stem THA due to other indications (femoral head necrosis, hip dysplasia, fracture or rheumatoid arthritis). The indication for THA and the preoperative Charnley classification did not significantly differ between both groups. There was also no significant difference in the rate of previous operations on the same hip between both groups.

**Table 1:** Demographic and clinical patient’s characteristics of the study cohort grouped for OB group and Non-OB group

Parameter	OB group (*n* = 69)	Non-OB group (*n* = 155)	*p* value
*Sex*			
Male	33 (47.8%)	74 (47.7%)	0.99
Female	36 (52.2%)	81 (52.3%)	
Age	60.0 ± 9.3	71.3 ± 11.0	0.40
Height (cm)	170.9 ± 11.1	170.7 ± 9.1	0.84
Weight (kg)	98.4 ± 15.3	75.9 ± 12.3	** < 0.001**
BMI (kg/m^2^)	33.5 ± 2.8	25.9 ± 2.6	** < 0.001**
*Side of THA*			
Right	40 (58.0%)	79 (51.0%)	0.33
Left	29 (42.0%)	76 (49.0%)	
*Indication for THA*			
Primary hip osteoarthritis	44 (63.8%)	106 (68.4%)	0.47
Secondary hip osteoarthritis	11 (15.9%)	29 (18.7%)	
Femoral head necrosis	7 (10.1%)	11 (7.1%)	
Hip dysplasia	6 (8.7%)	8 (5.2%)	
Fracture	1 (1.4%)	0	
Rheumatoid arthritis	0	1 (0.6%)	
*Charnley classification*			
A	36 (52.2%)	91 (58.7%)	0.45
B	28 (40.6%)	58 (37.4%)	
C	5 (7.2%)	6 (3.9%)	
Previous operation on the hip	6 (8.7%)	13 (8.4%)	0.94

Bold *p* values indicate statistically significant differences

Two patients (OB group: 0; Non-OB group: 2) had to be excluded at the 6 weeks–3 months follow-up (death: 1; withdrawn consent: 1) and ten patients (OB group: 4; Non-OB group: 6) missed the 6 weeks–3 months follow-up. Six patients (OB group: 2; Non-OB group: 4) had to be excluded at the 1-year follow-up (revision surgery with explanation: 2; withdrawn consent: 2) and six patients (OB group: 3; Non-OB group: 3) missed the 1-year follow-up. Five patients (OB group: 1; Non-OB group: 4) had to be excluded at the 2-year follow-up (withdrawn consent: 5) and six patients (OB group: 1; Non-OB group: 5) missed the 2-year follow-up. Twenty-one patients (OB group: 7; Non-OB group: 14) had to be excluded at the 5-year follow-up (death: 4; withdrawn consent: 17) and ten patients (OB group: 5; Non-OB group: 5) missed the 5-year follow-up. Ten patients (OB group: 4; Non-OB group: 6) had to be excluded at the 7-year follow-up (death: 3; withdrawn consent: 7) and fifteen patients (OB group: 4; Non-OB group: 11) missed the 7-year follow-up. Of the two patients, who underwent revision surgery with explanation of the optimys short stem and thus were excluded at the 1-year follow-up, one patient was in the OB group (revision because of a clinically relevant axial migration of the shaft) and one patient was in the Non-OB group (revision because of an periprosthetic infection). The mean follow-up period was 87.2 months (range 81.9–104.0 months).

### Operation characteristics

The operation characteristics of both groups are displayed in Table 2. The choice of the surgical approach for implantation of the short stem THA (anterolateral, direct anterior or lateral) did not significantly differ between both groups. In the Non-OB group, 31.6% of the performed THA implantation was rated by the surgeon as a minimally invasive procedure, whereas in the OB group, only 18.8% of the THA implantation were judged to be minimally invasive. Accordingly, a minimally invasive approach was performed significantly less frequently in obese patients (*p* = 0.049).

**Table 2:** Data on operation characteristics and length of hospital stay grouped for OB group and Non-OB group

Parameter	OB group (*n* = 69)	Non-OB group (*n* = 155)	*p* value
*Surgical approach*			
Anterolateral (Watson–Jones)	40 (58.0%)	96 (61.9%)	0.79
Direct anterior	16 (23.2%)	30 (19.4%)	
Lateral (Hardinge)	13 (18.8%)	29 (18.7%)	
*Minimally invasive*			
Yes	13 (18.8%)	49 (31.6%)	**0.049**
No	56 (81.2%)	106 (68.4%)	
Operating time (min)	71.9 ± 19.5	71.8 ± 18.6	0.81
Length of hospital stay (days)	9.9 ± 1.9	9.6 ± 2.4	0.21

Bold *p* value indicates statistically significant differences

The operating time (71.9 ± 19.5 min vs. 71.8 ± 18.6 min; *p* = 0.81) and the length of hospital stay after implantation of the short stem THA (9.9 ± 1.9 days vs. 9.6 ± 2.4 days; *p* = 0.21) showed no significant difference between both groups.

### General and specific complications

Table 3 lists general and specific complications grouped for the OB group and the Non-OB group. A total of two patients suffered a general complication perioperatively, including one patient in the OB group with an acute renal failure requiring pharmacological therapy and one patient in the Non-OB group with a myocardial infarction. The occurrence of general complications showed no significant difference when comparing the OB group and Non-OB group (1.4% vs. 0.6%; *p* = 0.55).

**Table 3:** Data on general and specific complications as well as axial migration after short stem THA grouped for OB group and Non-OB group

	OB group (*n* = 69)	Non-OB group (*n* = 155)	*p* value
Patients with a general complication	1 (1.4%)	1 (0.6%)	0.55
Patients with a specific complication	5 (9.7%)	15 (7.2%)	0.56
*Total number of specific complications*	7	18	
Irritation of N. cut. fem. lat	2	9	
Wound healing disorder/superficial surgical site infection	2	3	
Hematoma/seroma	0	2	
Dislocation	1	1	
Nerve paralysis	1	1	
Periprosthetic fracture of trochanter major	0	1	
Periprosthetic infection	0	1	
Patients with an axial migration > 1.5 mm	2 (2.9%)	6 (3.9%)	0.72

In the OB group, five patients suffered a total of seven specific complications within the 7 years of follow-up, including two irritations of the lateral femoral cutaneous nerve, two wound healing disorders/superficial surgical site infections, one dislocation, and one nerve paralysis. The dislocation occurred within the first 3 months after implantation and required a closed reduction but no surgical revision.

In the Non-OB group, 15 patients showed the following 18 specific complications within the 7 years of follow-up: 9 irritations of the lateral femoral cutaneous nerve, 3 wound healing disorders/superficial surgical site infections, 2 postoperative hematoma/seroma, 1 dislocation, 1 nerve paralysis, 1 periprosthetic fracture of the trochanter major, and 1 periprosthetic infection. The dislocation in the Non-OB group also occurred within the first 3 months after implantation and required a closed reduction but no surgical revision. The periprosthetic infection occurred within the first 3 months and needed a surgical revision with explanation of the short stem. According to the dropout criteria, this patient was excluded from the further follow-ups. The periprosthetic fracture of the trochanter major occurred between the 2-year and 5-year follow-up and an osteosynthetic treatment was performed in this case.

The rate of patients suffering a specific complication did not differ significantly between both groups (9.7% vs. 7.2%; *p* = 0.56).

A total of eight patients showed an axial migration above the threshold of 1.5 mm within the 7 years of follow-up. Of these eight patients, two patients belonged to the OB group and six patients belonged to the Non-OB group. One of the two patients from the OB group required a revision surgery with explanation of the short stem. The rate of patients with an axial migration > 1.5 mm was not significantly different between both groups (2.9% vs. 3.9%; *p* = 0.72).

### Revision surgery and implant survival

Within the 7 years of follow-up, a total of two revision surgeries with explanation of the optimys short stem were recorded. Both surgical revisions took place in the first 3 months postoperatively. The calculated overall survival rate of the optimys short stem within the 7 years of follow-up was 99.1%. The overall standardized revision rate was calculated to be 0.127, and thus the standardized revision rate was below the threshold of 1 indicating the safety and efficacy of the optimys short stem.

One of the two patients undergoing revision surgery belonged to the OB group and underwent revision surgery due to a clinically relevant axial migration of the short stem of 11 mm. The other patient belonged to the Non-OB group and required the surgical revision because of a periprosthetic infection. There was no significant difference for the rate of patients requiring a surgical revision between the OB group and the Non-OB group (1.4% vs. 0.6%; *p* = 0.55).

### Pain and patient satisfaction

**Fig. 2:**
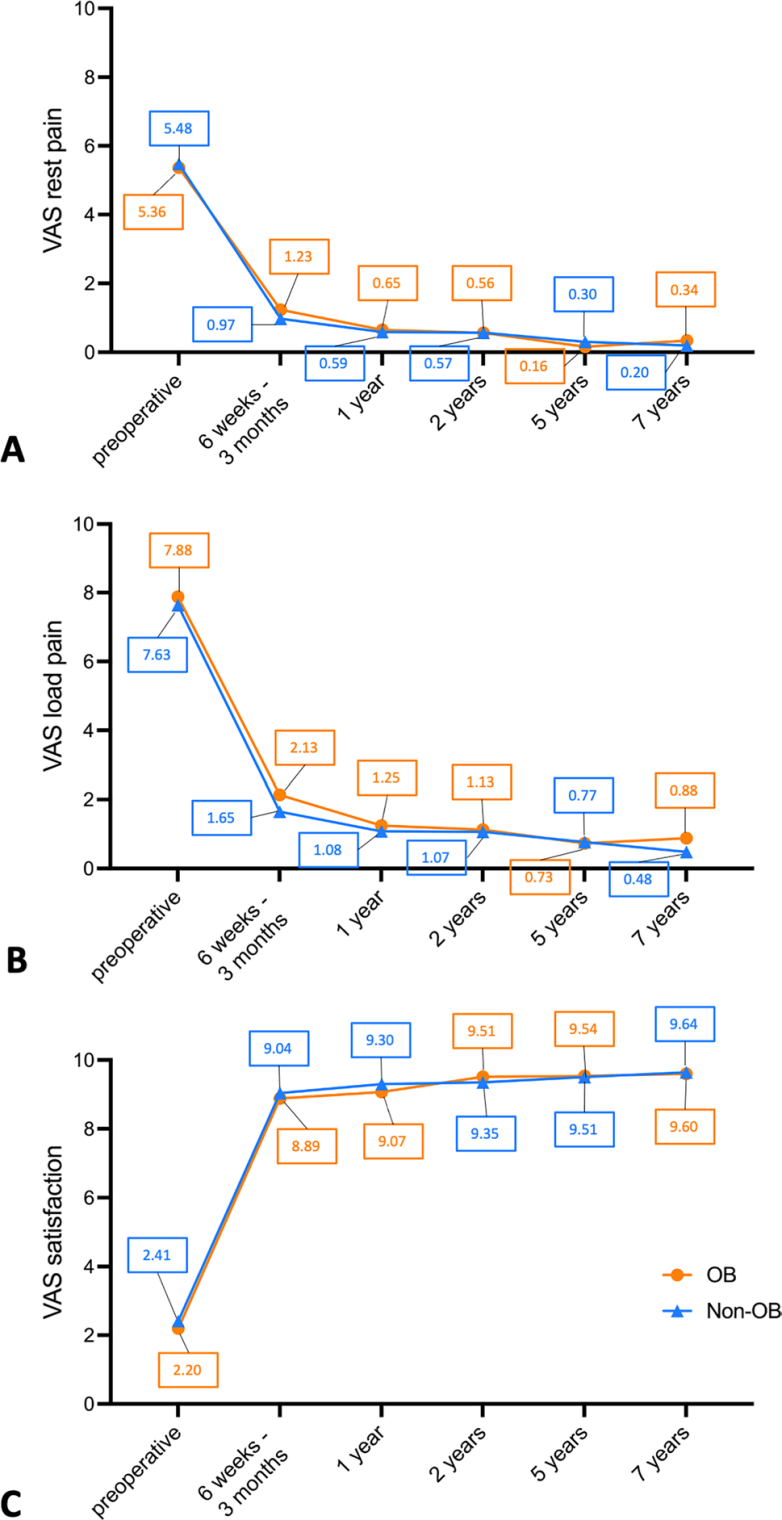
Changes over time of rest pain (**A**), load pain (**B**), and overall patient satisfaction (**C**), each according to the VAS scale. Data are grouped for OB group and Non-OB group. Symbols represent means, and the mean values from each follow-up visit are displayed within the plot

**Fig. 3:**
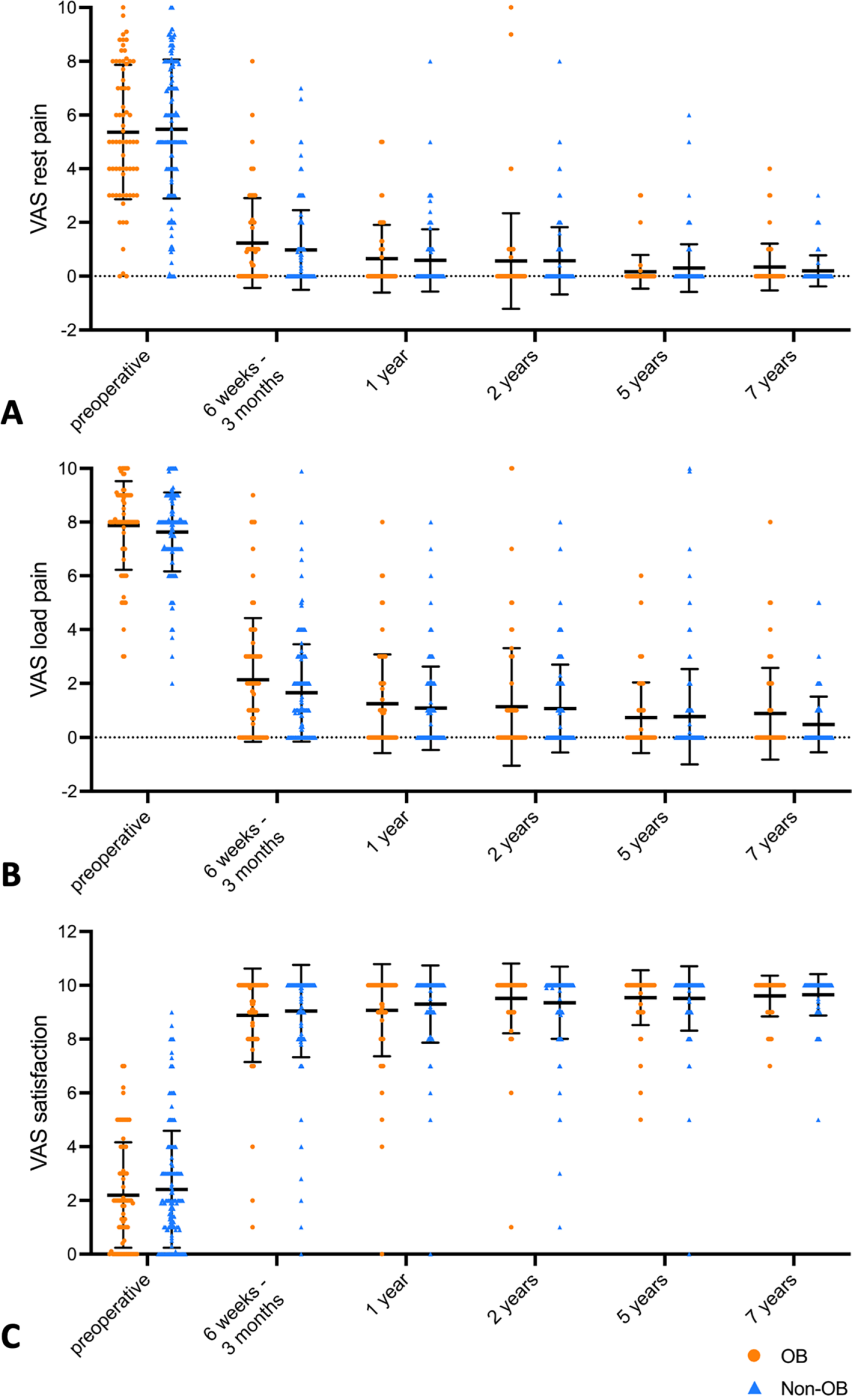
Dot plots of rest pain (**A**), load pain (**B**), and overall patient satisfaction (**C**), each according to the VAS scale. Data are grouped for OB group and Non-OB group. Lines represent median, whiskers represent standard deviation, and colored symbols represent individual values. Mann–Whitney-*U*-test was used for comparison. Exact *p* values are reported if *p* < 0.05

The preoperative rest pain (5.36 vs. 5.48; *p* = 0.64) and load pain (7.88 vs. 7.63; *p* = 0.06) based on the VAS scale did not significantly differ between the OB group and the Non-OB group (Figs. 2A + B, 3A + B). In both groups, rest pain and load pain improved continuously over the postoperative period (Fig. 2A + B). Only in the OB group, rest pain and load pain slightly increased again at the 7-year follow-up. When comparing the postoperative rest pain between the OB group and the Non-OB group, no significant difference could be observed at the 6 weeks–3 months (1.23 vs. 0.97; *p* = 0.18), the 1-year (0.65 vs. 0.59; *p* = 0.93), the 2-year (0.56 vs. 0.57; *p* = 0.42), the 5-year (0.16 vs. 0.30; *p* = 0.28), and the 7-year (0.34 vs. 0.20; *p* = 0.29) follow-up (Fig. 3A + B). Also, the postoperative load pain did not significantly differ between obese and non-obese patients at the 6 weeks–3 months (2.13 vs. 1.65; *p* = 0.29), the 1-year (1.25 vs. 1.08; *p* = 0.91), the 2-year (1.13 vs. 1.07; *p* = 0.66) the 5-year (0.73 vs. 0.77; *p* = 0.48), and the 7-year (0.88 vs. 0.48; *p* = 0.32) follow-up.

The overall patient satisfaction based on the VAS scale also improved continuously over the postoperative period in both groups (Fig. 2C). By comparing the overall satisfaction between obese and non-obese patients, no significant difference could be observed preoperatively (2.20 vs. 2.41; *p* = 0.66) as well as at the 6 weeks–3 months (8.89 vs. 9.04; *p* = 0.26), the 1-year (9.07 vs. 9.30; *p* = 0.43), the 2-year (9.51 vs. 9.35; *p* = 0.25), the 5-year (9.54 vs. 9.51; *p* = 0.96), and the 7-year (9.60 vs. 9.64; *p* = 0.68) follow-up (Fig. 3C).

### Harris Hip Score

**Fig. 4:**
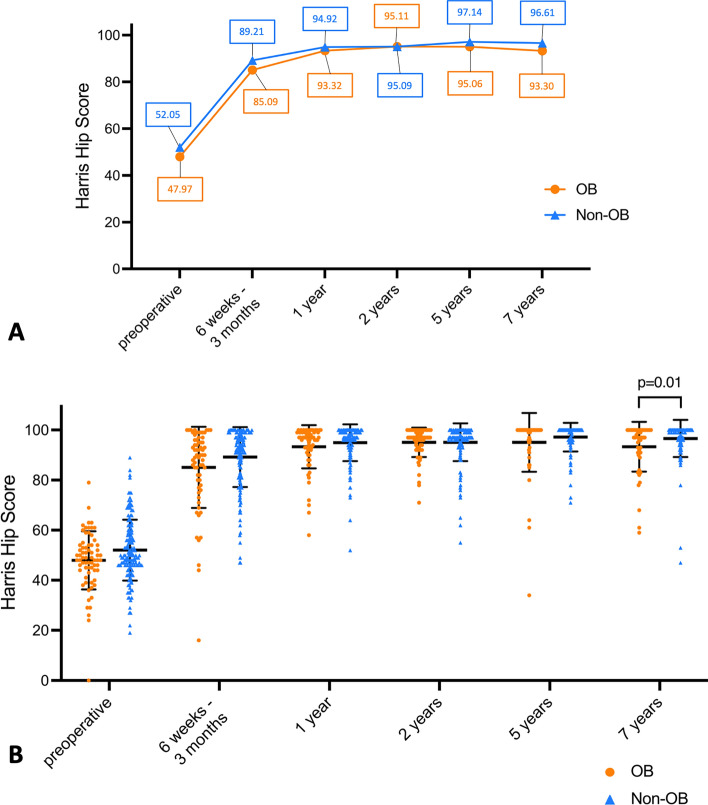
**A** Changes over time of Harris Hip Score from OB group and Non-OB group. Symbols represent means, and the mean values from each follow-up visit are displayed within the plot. **B** Dot plots of Harris Hip Score from OB group and Non-OB group. Lines represent median, whiskers represent standard deviation, and colored symbols represent individual values. Mann–Whitney-*U*-test was used for comparison. Exact *p* values are reported if *p* < 0.05

There was no significant difference of the preoperative HHS between obese and non-obese patients (47.97 vs. 52.05; *p* = 0.07) (Fig. 4B). In the OB group, the HHS improved postoperatively up to and including the 2-year follow-up and slightly decreased again at the 5-year and 7-year follow-up (Fig. 4A). In the Non-OB group, the HHS showed a postoperative improvement up to and including the 5-year follow-up with a slight decrease at the 7-year follow-up. The HHS was classified for both groups as good at the 6 weeks–3 months follow-up and as excellent at the 1-year, 2-year, 5-year, and 7-year follow-up. Furthermore, in both groups, the HHS was above the corresponding PASS threshold in each postoperative visit.

The postoperative HHS did not significantly differ between the OB group and the Non-OB group at the 6 weeks–3 months (85.09 vs. 89.21; *p* = 0.07), the 1-year (93.32 vs. 94.92; *p* = 0.11), the 2-year (95.11 vs 95.09; *p* = 0.50), and the 5-year (95.06 vs. 97.14; *p* = 0.86) follow-up (Fig. 4B). At the 7-year follow-up, the HHS of obese patients was significantly lower compared to the HHS of non-obese patients (93.30 vs. 96.61; *p* = 0.01).

## Discussion

Short stem THA, e.g., with the optimys stem, is a popular option in young patients, as these patients statistically will need at least one revision hip arthroplasty and the bone stock preserving effect of short stem THA will be beneficial for their revision hip arthroplasty [6, 8–14]. In this mid-term follow-up multicenter study about the effect of obesity on the outcome after short stem THA with the optimys prosthesis, we demonstrated that operating time, length of hospital stay, rate of general and specific complications, and rate of revision surgeries did not differ between obese and non-obese patients. In addition, VAS rest and load pain, VAS satisfaction, and the HHS improved from preoperatively to postoperatively in both groups after short stem THA. VAS rest and load pain as well as VAS satisfaction showed no significant difference between both groups, whereas the HHS of obese patients was significantly lower compared to non-obese patients only at the 7-year follow-up but still above the PASS threshold.

We further demonstrated that a minimally invasive approach was used significantly less often in obese patients compared to non-obese patients. A possible reason for this is the restricted visualization of and access to the operating area in obese patients when using a minimally invasive approach. Consistently, Argyrou et al. found an increased incision length in obese patients when performing a THA via a direct anterior approach [46]. However, minimally invasive approaches can be advantageous, since they were shown to improve patient satisfaction and early rehabilitation after THA as well as to lower the rate of wound complications [47–50]. Furthermore, several studies demonstrated that the choice of surgical approaches also affects the risk for infection in obese patients undergoing conventional THA [51, 52]. Hence, further studies are required to analyze the effect of different surgical approaches on complications and outcome after short stem THA in obese patients.

Several studies found an increased operating time for conventional THA in obese patients compared to non-obese patients, e.g., due to the lower visualization and access to the operative field [24–26, 28, 46, 53]. Luger et al. also demonstrated a longer operating time with about 80 min in severely obese patients (BMI ≥ 35 kg/m^2^) undergoing short stem THA with the Fitmore prosthesis via a minimally invasive anterolateral approach [34]. However, in our study using the optimys short stem, the operating time was not significantly increased in obese patients and amounted about 72 min in both groups, pointing out a potential advantage of the optimys short stem for obese patients.

In contrast to the literature about conventional THA reporting an increased length of hospital stay in obese patients [24, 54, 55], we could not observe a difference in the length of hospital stay after short stem THA between obese and non-obese patients. This is in line with the results of Luger et al. also reporting no difference in the length of hospital stay after short stem THA with the Fitmore prosthesis [34]. One reason for this can be the earlier rehabilitation after short stem THA, again highlighting the advantage of a short stem THA also for obese patients.

It is well studied that obesity significantly increases the rate of complications up to 4-fold after conventional THA. Especially the rates of wound infections (OR 2.71; increase of 89.5% in odds per 10 points increase in BMI), periprosthetic infections (OR 1.53; RR 2.92; increase of 61.3% in odds per 10 points increase in BMI), and prosthesis dislocations (OR 1.99; RR 2.08; increase of 113.9 in odds per 10 points increase in BMI) were found to be enhanced in obese patients compared to non-obese patients [24–28, 56, 57]. For short stem THA with the Fitmore prosthesis, Luger et al. observed that only severely obese patients (BMI ≥ 35 kg/m^2^) but not obese patients (BMI 30 to < 35 kg/m^2^) showed a higher risk for general surgical complications (OR 4.37) and especially for deep infections (OR 21.69), whereas obese and severely obese patients had no increased risk for dislocations [34]. In contrast, Chammai et al. observed comparable rates of complications in obese patients (7.3%) and non-obese patients (9.8%) undergoing short stem THA with the Metha prosthesis [35]. We did not observe a significant difference in the rate of specific complications between obese (9.7%) and non-obese patients (7.2%) undergoing short stem THA with the optimys short stem. In our study, there were two wound healing disorders/superficial infections in obese patients and three in non-obese patients, none periprosthetic infection in obese patients and one in non-obese patients as well as one dislocation in each of both groups. The main minor complication in our study cohort was an irritation of the lateral femoral cutaneous nerve (in two obese and nine non-obese patients) with a high rate of spontaneous recovery. Transient palsy of the lateral femoral cutaneous nerve was shown to be a typical complication after THA, especially in minimal invasive and anterior approaches, but was shown to improve with time and have no influence on the functional outcome [58–60].

Iwata et al. reported an increased risk of periprosthetic fractures of the greater trochanter in obese patients undergoing conventional THA via an anterolateral approach [61]. We could not confirm this finding, since we only found one periprosthetic fracture in the non-obese patient group at the 5-year follow-up. This is in line with a study of Luger et al., in which BMI was not a risk factor for periprosthetic fractures in short stem THA [33].

Besides an increased rate of complications, several studies observed a higher rate of revision surgeries in obese patients undergoing conventional THA with a revision rate of about 8%, an odds ratio of 1.61, and an increase of 52.4% in odds per 10 points increase in BMI. The most common reason for a revision was an infection [24, 27–30]. Luger et al. also reported an increased revision rate for severely (BMI 35 to < 40 kg/m^2^) and morbidly obese (BMI ≥ 40 kg/m^2^) patients undergoing short stem THA with the Fitmore prosthesis [34]. In our analysis of the optimys short stem, one revision per group had to be performed resulting in an overall revision rate of 0.9%. As a consequence, the optimys short stem had a survival rate of 99.1% within the 7 years of follow-up in our study, which is comparable to the reported survival rate of short stem prosthesis with 96–100% in the literature [15, 62, 63]. The overall standardized revision rate of 0.127 in our study was below the threshold of 1 indicating the safety of the optimys short stem [15].

Obese patients showed a slightly but not significantly increased axial migration of uncemented short stems compared to non-obese patients [17, 63–65]. Analysis of the axial migration of the optimys short stem was not an aim of this study, since the number of available postoperative X-rays for EBRA-FCA was too small. Nevertheless, we observed an axial migration above the threshold of 1.5 mm in two obese patients and six non-obese patients without a significant difference in the rate between both groups. However, one obese patient had to undergo revision surgery with explanation of the short stem prosthesis due to a clinically relevant axial migration of 11 mm. Consequently, axial migration of the optimys short stem in obese and non-obese patients should be the subject of further prospective studies, since an increased subsidence could lower the long-term survival, and thus can counteract the benefits of short stem THA.

There are only little data about the clinical outcome in obese patients after short stem THA so far. In conventional THA, pain improved from preoperatively to postoperatively and pain intensity did statistically not differ between obese and non-obese patients postoperatively [24, 66, 67]. In our study of the optimys short stem, rest pain and load pain were also improved from preoperatively to postoperatively without a significant difference between obese and non-obese patients over the entire follow-up period of 7 years. Kutzner et al. reported a rest pain of 0.1 and a load pain of 0.6 according to the VAS scale at the 5-year follow-up after short stem THA with the optimys prosthesis [62]. This is comparable to our findings at 5-year follow-up with a rest pain of 0.16 and 0.30 as well as a load pain of 0.73 and 0.77 in obese and non-obese patients, respectively.

Haebich et al. showed that obese patients exhibited a lower satisfaction than non-obese patients after conventional THA, whereas Goh et al. observed no significant effect of obesity on satisfaction [67, 68]. In our study about the optimys short stem, overall patient satisfaction according to the VAS scale was improved from preoperatively to postoperatively without a significant difference between obese and non-obese patients over the entire follow-up period of 7 years. The VAS patient satisfaction of 9.54 for obese patients and 9.51 for non-obese patients at the 5-year follow-up in our study is comparable to the VAS satisfaction of 9.7 at the 5-year follow-up in the study of Kutzner et al. about the optimys short stem [62].

The HHS after conventional THA was found to be significantly lower in obese patients compared to non-obese patients indicating a poorer functional outcome in obese patients undergoing THA in general [25, 26, 28, 57, 69]. Chammai et al. also observed a poorer HHS in obese patients after short stem THA with the Metha prosthesis compared to non-obese patients after a follow-up of about 50 months [35]. We here reported a postoperative improvement of the HHS after short stem THA with the optimys prosthesis in obese and non-obese patients without a significant difference between both groups up to and including the 5-year follow-up. Kutzner et al. recorded a HHS of 97.8 at 5 years after short stem THA with the optimys prosthesis [62], which is comparable to our findings with an HHS of 97.14 in non-obese patients and 95.06 in obese patients at the 5-year follow-up. The HHS observed in our patient cohort was higher than in the studies by Chammai et al. with 87.54 in obese and 92.49 in non-obese patients [35]. Interestingly, the HHS declined again at the 5-year and especially at the 7-year follow-up in the obese patient group in our study, so that the HHS was significantly lower in obese patients compared to non-obese patients at the 7-year follow-up. However, the HHS of obese patients at the 7-year follow-up (HHS 93.30) was still excellent and remained above the PASS threshold still indicating a good clinical function in the obese patient group after 7 years.

In sum, short stem THA with the optimys prosthesis provides a safe surgical procedure with a low complication rate, an excellent clinical function, a sufficient pain relief, and a good patient satisfaction in non-obese as well as obese patients. Therefore, this study contributes to a scientific basis for the expansion in indication for short stem THA also in obese patients. Nevertheless, measures to reduce body weight should still be recommended in obese patients before undergoing short stem THA. However, the effect of preoperative surgical and non-surgical interventions for weight loss on the outcome of THA is still unclear and conflicting, mainly due to a lack of high-quality, prospective, and randomized studies [70–74]. Thus, a holistic approach considering multiple individual risk factors of each patient and a shared decision-making should be used for short stem THA also in obese patients [75].

The following limitations of this study have to be mentioned. Although we prospectively analyzed 224 patients for this multicenter study based on our sample size calculation, the number of cases is rather small compared to other studies investigating complications and functional outcomes after THA. We included patients between 18 and 85 years in this study to match the patient collective, which receives a short stem prosthesis in the routine clinical practice. However, this can increase the heterogeneity of our study cohort, since patients’ activity levels can differ with age. Nevertheless, the mean age of both cohorts is comparable with other studies investigating the effect of obesity in short stem THA [33, 34, 36]. In addition, the multicenter study design can decrease the homogeneity of the study cohort because of different acetabular cups used and different surgeons who performed the implantation. On the other hand, the multicenter study design can in turn increase the diverse population coverage and the representativeness of the study cohort. Furthermore, analysis of axial migration was limited in this study, since the number of cases with radiological monitoring during the follow-up was too small. Studies focusing on investigation of axial migration of the optimys short stem in obese patients are further required, even though several studies already showed that axial migration of short stem prosthesis is not increased in obese patients [17, 63–65]. A follow-up period of 7 years is rather suitable to investigate the mid-term outcome [76]. While this study had already a longer follow-up period compared to other studies with mostly 4–5 years of follow-up, a continuation of this study is necessary to study the long-term outcome after 10–15 years.

## Conclusion

This study demonstrates that the rate for general and specific complications as well as the postoperative pain and patient satisfaction do not significantly differ between obese and non-obese patients undergoing short stem THA with the optimys prosthesis. Although HHS at 7-year follow-up was significantly lower in obese patients, an excellent HHS can be achieved in obese as well as non-obese patients from 1 year postoperative. Consequently, short stem THA with the optimys prosthesis is a safe and effective option also for obese patients with an excellent functional outcome. Obesity should not be a contraindication for short stem THA. However, measures for reduction of body weight should still be recommended in obese patients before THA.

## Data Availability

The datasets used and/or analyzed during this study are available from the corresponding author on reasonable request.
